# Ferritinophagy Loss Drives Mitochondrial Iron Import and Colorectal Tumorigenesis

**DOI:** 10.21203/rs.3.rs-7483419/v1

**Published:** 2025-09-23

**Authors:** Xiang Xue, Hyeoncheol Kim, Luke Villareal, Naiara Santana-Codina, Christian Cabanlong, Lavanya Goodla, Eric Prossnitz, David Martin, Joseph Mancias

**Affiliations:** University of New Mexico; University of New Mexico; UNM; Dana Farber Cancer Institute; University of New Mexico; University of New Mexico; University of New Mexico; University of New Mexico; Dana Farber Cancer Institute

**Keywords:** Iron Metabolism, Colorectal Carcinogenesis, Reactive Oxygen Species

## Abstract

Iron is an essential cofactor for mitochondrial metabolism, yet the regulatory networks linking cellular iron homeostasis to colorectal cancer (CRC) progression remain incompletely understood. Here, we identify nuclear receptor coactivator 4 (NCOA4), a ferritinophagy receptor, as a context-dependent tumor suppressor that coordinates cytosolic and mitochondrial iron handling in CRC. Analysis of human tumors and colon-specific *Ncoa4* knockout mice revealed that NCOA4 loss drives tumorigenesis by inducing transferrin receptor–mediated iron uptake and mitochondrial calcium uniporter (MCU)–dependent mitochondrial iron import. This dual iron overload elevates mitochondrial reactive oxygen species, activates STAT3 signaling, and enhances tumor cell proliferation. NCOA4 overexpression reverses these effects, reducing MCU expression and tumor growth. Pharmacological inhibition of MCU, STAT3, or mitochondrial iron transport mitigated tumorigenesis in NCOA4-deficient models. Our findings define an NCOA4–MCU–STAT3 metabolic signaling axis that couples iron metabolism to oncogenic progression and reveal mitochondrial iron handling as a therapeutic vulnerability in CRC.

## Introduction

Colorectal cancer (CRC) is a major global health problem and a leading cause of cancer-related deaths in the United States (Rawla et al., 2019). By 2040, CRC is projected to have the highest global incidence among all cancers, driven by population aging, growth, and lifestyle-related risk factors (Soerjomataram et al., 2021). Although U.S. incidence declined for decades due to screening and preventive measures, CRC remains the third most common cancer and the third leading cause of cancer death (Siegel et al., 2023). In 2023, an estimated 153,020 new cases and 52,550 deaths were reported, with annual treatment costs expected to reach $21 billion by 2030 (Stukalin et al., 2022). Despite medical advances, survival for advanced CRC remains poor, underscoring the need for novel therapeutic strategies.

Iron, an essential dietary micronutrient, has been linked to CRC risk. Epidemiological studies show that high iron levels increase CRC risk, whereas iron reduction decreases it (Lee et al., 2004; Osborne et al., 2010; Zacharski et al., 2008). Human and mouse colon tumors exhibit elevated iron compared to normal tissue (Pusatcioglu et al., 2014; Xue et al., 2016), and dietary iron restriction reduces tumor burden in preclinical models (Xue et al., 2012), while high iron intake promotes growth (Radulescu et al., 2012). However, iron deficiency anemia in many CRC patients limits systemic iron deprivation as a therapy.

Mitochondria are central to iron metabolism, producing iron–sulfur clusters and driving ROS generation that supports tumor proliferation (Rouault et al., 2005; Dixon et al., 2014). We previously showed that targeting mitochondrial iron with the FDA-approved chelator deferiprone or reducing ROS with TEMPO suppresses colon tumorigenesis without affecting systemic iron (Xue et al., 2017), highlighting mitochondrial iron metabolism as a therapeutic target.

Ferritinophagy, mediated by nuclear receptor coactivator 4 (NCOA4), regulates iron release from ferritin for cellular use (Mancias et al., 2014). It plays critical roles in erythropoiesis (Santana-Codina et al., 2019; Ryu et al., 2017) and ferroptosis—an iron-dependent cell death pathway (Dixon et al., 2012; Yu et al., 2022). Dysregulated ferritinophagy contributes to cancer, including therapy resistance in pancreatic tumors via enhanced mitochondrial iron delivery (Jain et al., 2022). NCOA4’s effects on iron metabolism are tissue-specific, as whole-body knockout reduces systemic iron but increases tissue iron in the liver, spleen, and intestines (Bellelli et al., 2016). Liver-specific NCOA4 knockdown increases hepatic iron levels, while overexpression reduces them (Li et al., 2020; Li et al., 2022). However, its role in colonic iron homeostasis and CRC remains unclear.

Here, we show that NCOA4 expression is reduced in human CRC and that colon-specific NCOA4 deletion in mice accelerates tumorigenesis, whereas overexpression suppresses it. NCOA4 deficiency caused cytosolic and mitochondrial iron accumulation, increased ROS, and enhanced TFRC-mediated iron uptake. Proteomics revealed upregulation of the mitochondrial calcium uniporter (MCU), promoting mitochondrial iron loading, ROS production, and activation of oncogenic STAT3 signaling (Xue et al., 2016). These findings identify NCOA4 as a key regulator of iron metabolism and CRC progression, with potential as a therapeutic target.

## Results

### NCOA4 is decreased in colon tumors and predicts CRC patient survival

**Figure 1:**
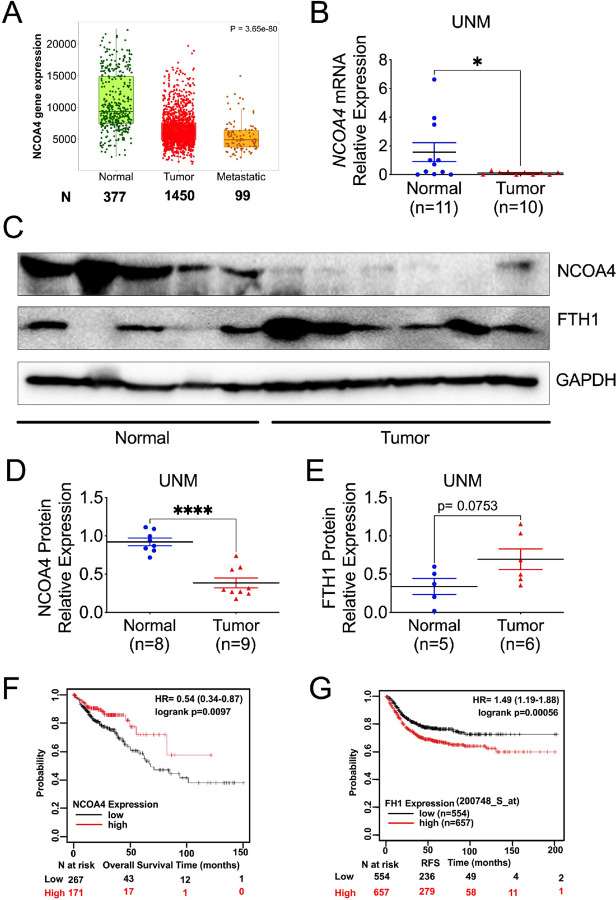
NCOA4 is decreased in colon tumors and predicts CRC patient survival. (**A**) NCOA4 mRNA expression in human normal (n = 377), tumor (n = 1450), and metastatic (n = 99) colon tissues from the TNMplot database. (**B**) qPCR analysis of colorectal tumors (n = 11) and their adjacent normal colon tissues (n = 10). (**C**) Immunoblotting analysis and quantification of (**D**) NCOA4 and (**E**) FTH1 protein levels from colorectal tumors and adjacent normal colon tissues. Kaplan–Meier survival curves of (**F**) NCOA4 and (**G**) FTH1 gene expression in colorectal tumor patients using data from the KMPlotter database. RFS, relapse-free survival. *p < 0.05, ****p < 0.0001. Unpaired Student’s t-test for B, D, and E.

Bioinformatic analysis revealed that *NCOA4* expression is significantly reduced in human colon tumors, with the lowest levels observed in metastatic tissues (Fig. 1A). Consistent with these findings, our independent analysis of colorectal cancer (CRC) samples from our institution demonstrated a similar downregulation of *NCOA4* mRNA in tumor tissues compared with normal colonic mucosa (Fig. 1B). Immunoblotting confirmed reduced NCOA4 protein levels in tumor samples (Figs. 1C, 1D), whereas FTH1—a marker of NCOA4 inactivation (Santana-Codina and Mancias, 2018)—was moderately upregulated in the same samples (Figs. 1C, 1E). Reduced *NCOA4* mRNA expression has been associated with poorer cancer patient survival (Gu et al., 2022). Analysis of a colorectal adenocarcinoma dataset (TCGA, PanCancer Atlas) in cBioPortal (Cerami et al., 2012) revealed that ~ 2.5% of CRC patients (13/526) harbor *NCOA4* mutations (missense, truncating, or deep deletions) (**Fig. S1A**). Progression-free survival was significantly lower in patients with *NCOA4* mutations compared to those without (p = 0.0102) (**Fig. S1B**). Kaplan–Meier survival curves from the KMPlotter database similarly indicated that low *NCOA4* expression is associated with poorer overall survival in CRC patients (Fig. 1F), and high FTH1 expression correlated with unfavorable prognosis (Fig. 1G). Together, these findings demonstrate that *NCOA4* downregulation, accompanied by FTH1 accumulation, is associated with colorectal tumorigenesis and poor clinical outcomes, supporting a critical role for NCOA4 in CRC pathophysiology.

Under iron-replete conditions, NCOA4 undergoes proteasomal degradation (Mancias et al., 2015). We observed reduced NCOA4 levels in colon tumors from *Cdx2*^Cre-ERT2^ *Apc*^F/+^ mice mice (Xue et al., 2016) fed a normal chow diet, as well as in normal colon tissues of mice fed a high-iron diet (**Figs. S1C, S1D**). These results suggest that NCOA4 downregulation is conserved between humans and mice, validating mouse models as relevant systems for studying human colon tumorigenesis.

### Colon-specific NCOA4 deficiency promotes tumor development in mouse models of CRC

To investigate the role of NCOA4 in colon tumorigenesis, we generated *Cdx2*^ERT2-Cre^ *Ncoa4*^F/F^ mice, enabling colon epithelial cell–specific deletion of *Ncoa4*. Using a well-established colitis-associated cancer (CAC) model induced by azoxymethane (AOM) and dextran sodium sulfate (DSS) (Thaker et al., 2012; Lee et al., 2022), we found that both tumor number and burden were significantly increased in *Cdx2*^ERT2-Cre^ *Ncoa4*^F/F^ mice following tamoxifen (TAM) administration to activate Cre recombinase (**Figs. S2A–S2C**). Histological analysis with hematoxylin and eosin (H&E) staining revealed increased high-grade dysplasia in *Ncoa4*-deficient colons compared with controls (**Figs. S2D, S2E**). Immunofluorescence analysis showed increased Ki-67 expression (**Figs. S2D, S2F**), indicating enhanced proliferation, while cleaved caspase-3 (CC3), a marker of apoptosis, showed no significant change (**Figs. S2D, S2G**).

**Figure 2:**
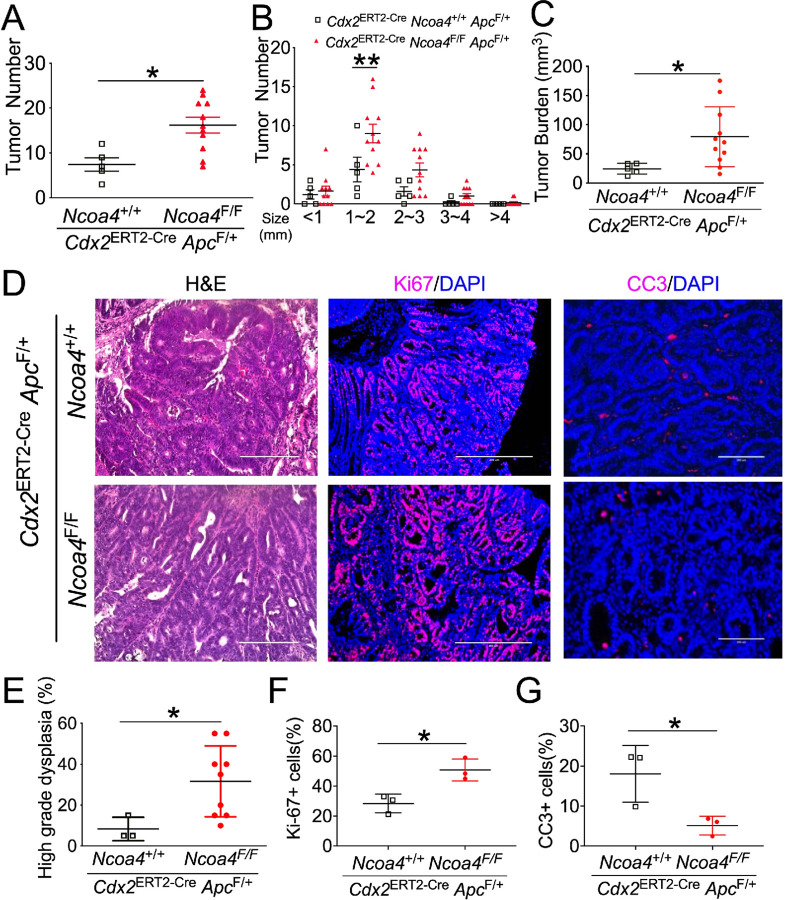
Colon-specific NCOA4 knockout increases colon tumorigenesis. (**A**) Tumor number, (**B**) tumor number by size, (**C**) tumor burden, (**D**) representative histological images, and quantification of (**E**) high-grade dysplasia, (**F**) Ki-67+ cells, and (**G**) cleaved caspase 3 (CC3)+ cells in colon tumors from *Cdx2*^ERT2-Cre^ *Ncoa4*^F/F^ *Apc*^F/+^ (n = 3–11) and *Cdx2*^ERT2-Cre^ *Ncoa4*^+/+^ *Apc*^F/+^mice (n = 3–5). *p < 0.05, **p < 0.01. Unpaired Student’s t-test for A, C, E–G. Two-way ANOVA with Sidak’s multiple comparisons test for B.

To further validate these findings, we generated mice with monoallelic *Apc* deletion (*Cdx2*^ERT2-Cre^ *Ncoa4*^F/F^ *Apc*^F/+^ and control *Cdx2*^ERT2-Cre^ *Ncoa4*^+/+^ *Apc*^F/+^). These mice develop colorectal tumors following repeated DSS exposure. After TAM-induced recombination and DSS treatment, tumor number and burden were significantly elevated in *Ncoa4*-deficient *Apc*^F/+^ mice compared with controls (Figs. 2A–2C). H&E staining confirmed more extensive high-grade dysplasia in *Cdx2*^ERT2-Cre^ *Ncoa4*^F/F^ *Apc*^F/+^ mice (Figs. 2D, 2E). Immunofluorescence again showed increased Ki-67 and reduced CC3 (Figs. 2D, 2F, 2G). Collectively, results from two independent models—AOM/DSS-induced CAC and monoallelic *Apc* deletion—demonstrate that NCOA4 loss promotes colon tumorigenesis, likely by facilitating tumor initiation and progression through increased epithelial proliferation and dysplastic transformation.

### Ncoa4 knockout increases intracellular iron, ATP levels, and xenograft tumor growth

**Figure 3:**
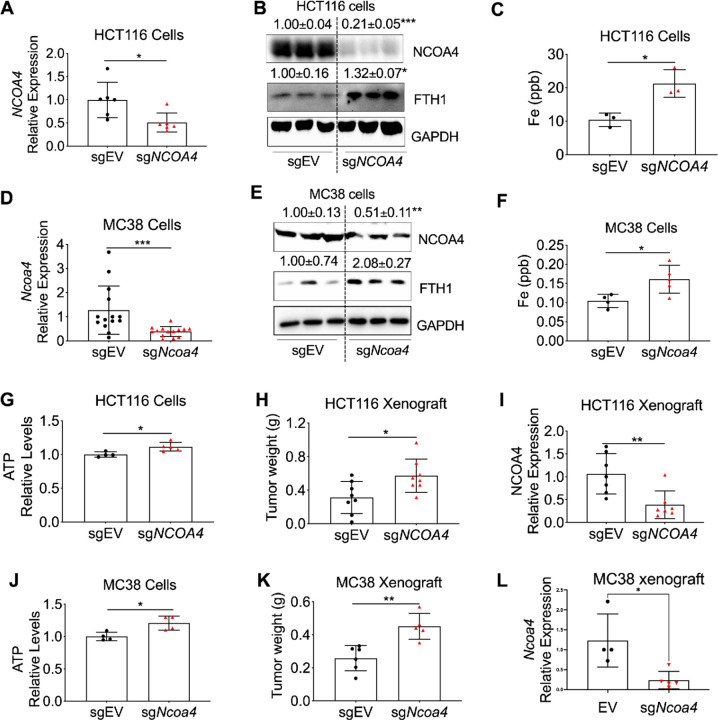
NCOA4 knockout increases intracellular iron, ATP levels, and xenograft tumor growth in CRC cells. (**A**) qPCR, (**B**) immunoblotting, and (**C**) ICP-MS analysis of iron levels in HCT116 cells (n = 3–6) with NCOA4 knockout (sg*NCOA4*) or wild-type control (sgEV). (**D**) qPCR, (**E**) immunoblotting, and (**F**) ICP-MS analysis of iron levels in MC38 cells (n = 3–14) with Ncoa4 knockout (sg*Ncoa4*) or wild-type control (sgEV). (**G**) CellTiter-Glo assay for HCT116 sgEV and sg*NCOA4* cells. (**H**) Tumor weight and (**I**) NCOA4 expression in xenograft tumors derived from HCT116 sgNCOA4 (n = 5–8) or sgEV (n = 4–8) cells. (**J**) CellTiter-Glo assay for MC38 sgEV and sg*Ncoa4* cells. (**K**) Tumor weight and (**L**) Ncoa4 expression in xenograft tumors derived from MC38 sg*Ncoa4* (n = 4–5) or sgEV (n = 4–6) cells. Values above blots are mean ± S.D. *p < 0.05, **p < 0.01, ***p < 0.001. Unpaired Student’s t-test.

Given that *Ncoa4* deletion enhances colon tumor formation *in vivo*, we next examined its effects on CRC cell behavior and tumor progression. *NCOA4* was stably knocked out in human HCT116 cells, confirmed by qPCR (Fig. 3A) and immunoblotting (Fig. 3B). Consistent with previous studies (Bellelli et al., 2016), NCOA4 knockout resulted in FTH1 upregulation (Fig. 3B). Total intracellular iron levels were significantly elevated in knockout cells, as measured by inductively coupled plasma mass spectrometry (ICP-MS; Fig. 3C). Similarly, *Ncoa4* deletion in mouse MC38 CRC cells led to increased FTH1, elevated intracellular iron (Figs. 3D–3F), increased ATP levels (Figs. 3G, 3J), and enhanced tumor growth *in vivo* (Figs. 3H, 3K), with NCOA4 downregulation confirmed in each case (Figs. 3I, 3L). Collectively, these results show that NCOA4 loss causes intracellular iron overload, promoting higher ATP production and tumor growth.

### NCOA4 depletion increases iron uptake–related but not autophagy-related proteins in colon tumors

NCOA4 functions both as a canonical autophagy receptor and a driver of ferritin condensate formation, which are degraded via macroautophagy and endosomal microautophagy (Ohshima et al., 2022). Since autophagy supports mitochondrial function by regulating iron metabolism in pancreatic cancer (Mukhopadhyay et al., 2023), we examined whether autophagy contributes to enhanced colon tumor growth in the context of *Ncoa4* deficiency.

Immunoblotting showed that *Ncoa4* deletion did not alter key autophagy-related proteins (ATG5, ATG16L, p62, LC3-II/I, p-S6K) in colon tumors (**Fig. S3A**). Furthermore, treatment with the autophagy inhibitor chloroquine failed to suppress increased xenograft tumor growth derived from MC38 sg*Ncoa4* cells in C57BL/6 mice (**Fig. S3B**), suggesting that neither canonical autophagy nor ferritinophagy primarily drive tumor growth following *Ncoa4* loss. This aligns with previous reports indicating ferritinophagy is not required for colon cancer cell growth (Hasan et al., 2020).

**Figure 4:**
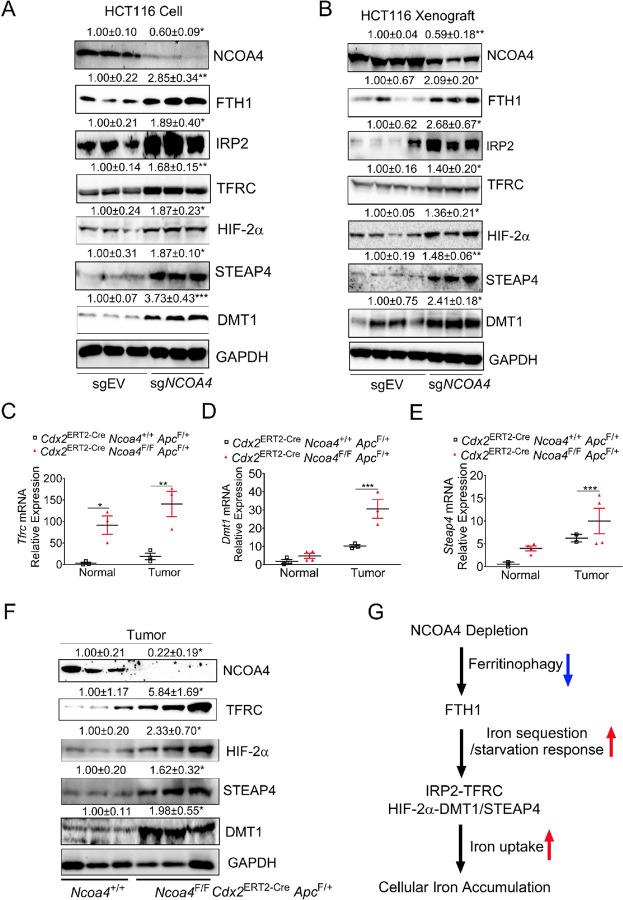
NCOA4 depletion induces iron uptake machinery in CRC. Immunoblotting analysis in (**A**) HCT116 sgEV and sg*NCOA4* cells and (**B**) their derived xenografts. qPCR analysis of (*C*) Tfrc, (**D**) Dmt1, and (**E**) Steap4, and (**F**) immunoblotting analysis for colon tissues from *Cdx2*^ERT2-Cre^ *Ncoa4*^F/F^ *Apc*^F/+^mice (n = 3) and *Cdx2*^ERT2-Cre^ *Ncoa4*^+/+^ *Apc*^F/+^mice (n = 3). (**G**) Proposed mechanism: NCOA4 depletion represses ferritinophagy and stabilizes FTH1, causing iron sequestration in ferritin and mimicking iron starvation. This induces IRP2-TFRC and HIF-2α-DMT1/STEAP4 signaling, increasing cellular iron uptake and cytosolic free iron. Values above blots are mean ± S.D. *p < 0.05, **p < 0.01, ***p < 0.001. Unpaired Student’s t-test for A, B, and F. Two-way ANOVA with Sidak’s multiple comparisons test for C–E.

In contrast, iron regulatory protein 2 (IRP2) and its downstream effector transferrin receptor (TFRC)—key mediators of cellular iron uptake (Kim et al., 2023; Zhao et al., 2024)—were significantly upregulated in *Ncoa4*-deficient cells and xenograft tumors (Figs. 4A, 4B). Hypoxia-inducible factor 2α (HIF-2α) and its downstream targets, Six-Transmembrane Epithelial Antigen of the Prostate 4 (STEAP4) and divalent metal transporter 1 (DMT1)—which promote a pro-tumorigenic iron-dependent state (Cheng et al., 2013; Xue et al., 2016, 2017)—were also elevated in *Ncoa4* knockout models (Figs. 4A, 4B). qPCR analysis confirmed increased *Tfrc*, *Dmt1*, and *Steap4* expression in colon tumors from *Cdx2*^ERT2-Cre^ *Ncoa4*^F/F^ *Apc*^F/+^ mice (Figs. 4C–4E). Immunoblotting corroborated increased TFRC, HIF-2α, DMT1, and STEAP4 proteins in these tumors (Fig. 4F).

Since NCOA4 was initially characterized as an androgen receptor (AR) coactivator (Yeh et al., 1996), and AR protein negatively correlates with TFRC levels in human CRC (Firehose Legacy dataset; **Fig. S3C**), we hypothesized AR antagonism might influence TFRC expression. However, treatment with the AR antagonist bicalutamide repressed TFRC protein at high doses (**Fig. S3D**), suggesting AR signaling does not significantly mediate *Ncoa4* deficiency–induced tumor growth.

Together, these data support a model whereby *Ncoa4* depletion suppresses ferritinophagy and stabilizes FTH1, causing iron sequestration in ferritin that mimics iron starvation. This triggers IRP2–TFRC and HIF-2α pathways to enhance iron uptake and accumulation in CRC (Fig. 4G), promoting tumorigenesis through pro-tumorigenic iron signaling.

### TFRC depletion abolishes NCOA4 deficiency–enhanced colon tumorigenesis

Given the critical role of iron accumulation mediated by the IRP2/TFRC axis in CRC (Lee et al., 2004; Shen et al., 2018; Yang et al., 2022; Kim et al., 2023), we tested whether inhibiting iron uptake could mitigate tumorigenesis induced by *Ncoa4* deletion. Genetic knockout of *Tfrc* in *Cdx2*^ERT2-Cre^ *Ncoa4*^F/F^ *Apc*^F/+^ mice abolished tumor formation driven by *Ncoa4* loss (**Figs. S4A–S4C**). H&E and immunofluorescence staining showed that increased proliferation (Ki-67) observed in *Ncoa4*-deficient tumors was reversed by *Tfrc* deletion (**Figs. S4D, S4E**). Conversely, apoptosis marker cleaved caspase-3 (CC3) was unchanged in *Ncoa4*-deficient tumors but significantly elevated in *Ncoa4*/*Tfrc* double knockout tumors (**Figs. S4D, S4F**). These findings demonstrate that TFRC upregulation is essential for *Ncoa4* deletion–induced colorectal tumorigenesis.

### Ncoa4 knockout robustly increases MCU mRNA and protein levels in mouse colon tissues

**Figure 5:**
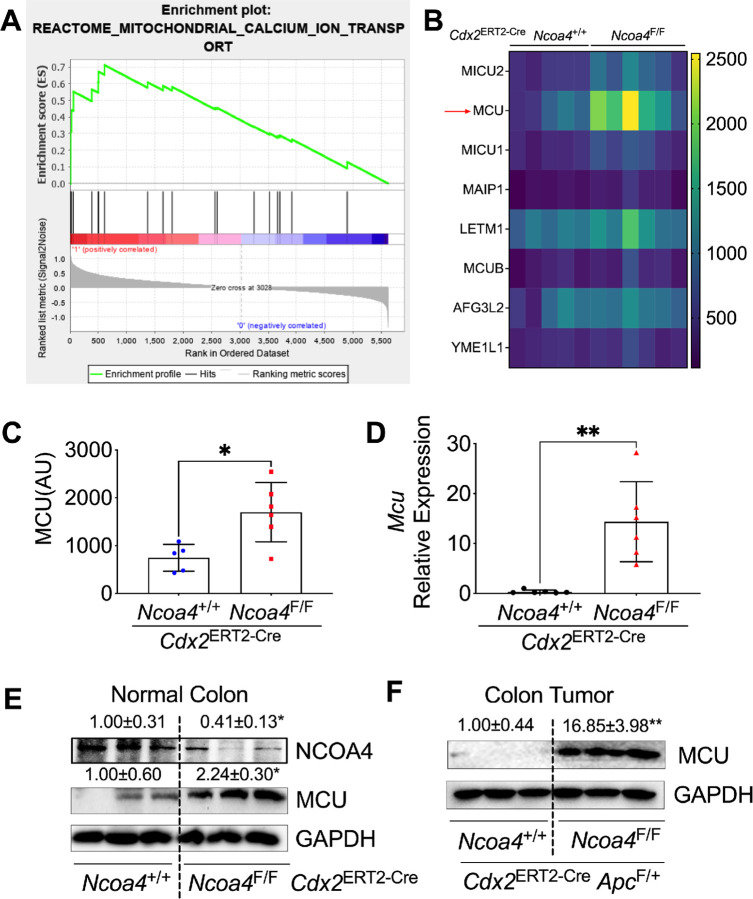
NCOA4 knockout robustly increases mRNA and protein levels of mitochondrial calcium uniporter (MCU) in mouse colon tissues. (**A**) Gene set enrichment analysis from unbiased quantitative proteomics showing enrichment of mitochondrial calcium ion transport proteins. (**B**) Heatmap of enriched mitochondrial calcium ion transport proteins. (**C**) Quantitative proteomics values for MCU. (**D**) qPCR and (**E**) immunoblotting analysis of colon tissues from colon epithelial cell-specific NCOA4 knockout mice (*Cdx2*^ERT2-Cre^ *Ncoa4*^F/F^, n = 3–6) and wild-type controls (*Cdx2*^ERT2-Cre^ *Ncoa4*^+/+^, n = 3–6). (**F**) Immunoblotting analysis of colon tumor tissues from *Cdx2*^ERT2-Cre^ *Ncoa4*^F/F^ *Apc*^F/+^ mice (n = 3) and wild-type controls (n = 3). *p < 0.05, **p < 0.01. Unpaired Student’s t-test.

To explore downstream effects of iron accumulation following *Ncoa4* deletion, we performed unbiased quantitative proteomics comparing colon tissues from *Cdx2*^ERT2-Cre^ *Ncoa4*^+/+^ and *Cdx2*^ERT2-Cre^ *Ncoa4*^F/F^ mice (**Table S1**). Metascape Enriched Ontology Clustering (**Fig. S5A**) and Gene Set Enrichment Analysis (Fig. 5A) revealed robust upregulation of mitochondrial proteins, particularly those involved in mitochondrial calcium ion transport. Volcano plot (**Fig. S5B**) and heatmap (Figs. 5B, 5C) analyses identified the mitochondrial calcium uniporter (MCU) as one of the most significantly increased proteins. qPCR (Fig. 5D) and immunoblot (Fig. 5E) analyses confirmed significantly elevated *Mcu* mRNA and MCU protein levels in colon tissues from *Cdx2*^ERT2-Cre^ *Ncoa4*^F/F^ mice versus controls. MCU protein was also markedly increased in colon tumors from *Cdx2*^ERT2-Cre^ *Ncoa4*^F/F^ *Apc*^F/+^ mice compared to controls (Fig. 5F). These data indicate that *Ncoa4* knockout induces MCU upregulation in the colon.

### AMPK–CREB signaling pathway is required for MCU induction in NCOA4 knockout cells

**Figure 6:**
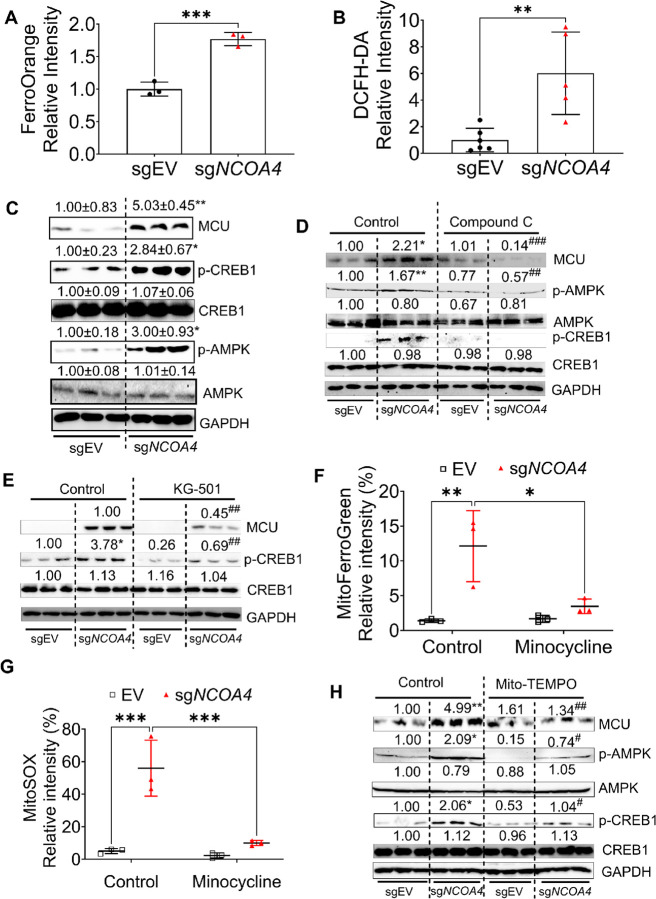
AMPK-CREB signaling pathway is required for MCU induction in NCOA4 knockout cells. (**A**) FerroOrange staining quantification, (**B**) DCFH-DA staining, and (**C**) immunoblotting analysis in human HCT116 sg*NCOA4* (n = 3) and sgEV control cells (n = 3). Immunoblotting analysis of HCT116 sg*NCOA4*and sgEV cells treated with (**D**) AMPK inhibitor compound C or (**E**) CREB inhibitor KG-501. (**F**) Mitochondrial iron measured by MitoFerroGreen staining and (**G**) mitochondrial ROS measured by MitoSOX staining in HCT116 sg*NCOA4* and sgEV cells treated with DMSO or MCU inhibitor minocycline. (**H**) Immunoblotting analysis of HCT116 sg*NCOA4* and sgEV cells treated with or without mitochondrial ROS inhibitor mito-Tempol. Values above blots are mean ± S.D. or means only. *p < 0.05, **p < 0.01, ***p < 0.001 vs. untreated sgEV; #p < 0.05, ##p < 0.01, ###p < 0.001 vs. untreated sgNCOA4. Unpaired Student’s t-test for A–C. Two-way ANOVA with Sidak’s multiple comparisons test for D–H.

MCU expression is transcriptionally regulated by phosphorylated CREB1 (Takahashi et al., 2019; Shanmughapriya et al., 2015), which is modulated by phosphorylated AMPK. AMPK activity is influenced by reactive oxygen species (ROS) (Thomson et al., 2008; Hwang et al., 2014; Agostini et al., 2023). Since iron catalyzes ROS production via Fenton chemistry, we assessed intracellular iron and ROS in HCT116 sg*NCOA4* cells. Both intracellular iron (Fig. 6A) and total ROS (Fig. 6B) were elevated. Proteins involved in the p-AMPK/p-CREB1/MCU axis were upregulated in *NCOA4*-deleted cells (Fig. 6C). Treatment with the AMPK inhibitor Compound C decreased p-AMPK, p-CREB1, and MCU levels in human and mouse *Ncoa4*-deficient cells (Figs. 6D, **S4C**). Similarly, the CREB1 inhibitor KG-501 suppressed p-CREB1 and MCU expression (Figs. 6E, **S5D**). MCU regulates mitochondrial calcium uptake and metabolism during CRC progression (Zeng et al., 2018; Liu et al., 2020) and mediates mitochondrial import of divalent metals including manganese and iron (Wettmarshausen et al., 2018; Sripetchwandee et al., 2014; Arcos et al., 2025). Increased MCU expression in *NCOA4*-deficient cells suggests enhanced mitochondrial iron import, elevating mitochondrial ROS (mtROS) (Xue et al., 2017). Indeed, both mitochondrial iron and mtROS were elevated in *NCOA4* knockout cells but were reduced by the MCU inhibitor minocycline (Schwartz et al., 2013; Hu et al., 2024; Arcos et al., 2025) (Figs. 6F, 6G). Treatment with the mitochondrial ROS scavenger mito-tempo decreased mtROS and downregulated p-AMPK, p-CREB1, and MCU protein levels (Fig. 6H). These data support a model wherein *NCOA4* loss promotes intracellular iron accumulation and ROS production, activating AMPK–CREB1 signaling, which induces MCU expression and further mitochondrial iron and ROS accumulation (**Fig. S5E**).

### NCOA4 deletion alters oxidative stress response in colon tumors and cells

We observed that the master antioxidant regulator nuclear factor erythroid 2-related factor 2 (NRF2) downstream targets—Heme Oxygenase-1 (HO-1) and NAD(P)H:quinone oxidoreductase 1 (NQO1)—were significantly reduced in colon tumors from *Ncoa4* knockout mice, while Kelch-like ECH-associated protein 1 (KEAP1) levels remained unchanged (**Fig. S6A**). Similar reductions in HO-1 and NQO1 were seen in MC38 *Ncoa4* knockout cells (**Fig. S6B**). Consistently, human NQO1 antioxidant response element (ARE) luciferase reporter activity was decreased in MC38 *Ncoa4* knockout cells (**Fig. S6C**). Markers of oxidative stress, including lipid peroxidation marker 4-hydroxynonenal (4-HNE), NQO1, glutathione peroxidase 4 (GPX4), and the cystine/glutamate antiporter xCT were elevated *in vitro* (**Fig. S6D**). In xenograft tumors, only xCT was reduced significantly, while other oxidative stress markers showed no changes (**Fig. S6E**). This discrepancy may reflect the presence of dietary vitamin E, a lipid peroxidation scavenger abundant in chow diets but absent in culture media. Vitamin E is known to protect against lipid peroxidation and ferroptosis (Hu et al., 2021). These findings suggest that increased proliferation in *Ncoa4*-deficient cells is balanced by enhanced lipid peroxidation–induced ferroptosis *in vitro*. *In vivo*, ferroptosis may be attenuated by antioxidant mechanisms such as dietary vitamin E. Supporting this, feeding mice a vitamin E-deficient diet for one week abolished the tumor-promoting effect of *Ncoa4* knockout (**Figs. S6F, S6G**). These data implicate ferroptosis sensitivity in the tumorigenic effects of *Ncoa4* deficiency (Anandhan et al., 2023).

### NCOA4 deletion activates STAT3 signaling via MCU-mediated mitochondrial iron accumulation

Iron accumulation can activate STAT3 via phosphorylation (Xue et al., 2016). Consistent with this, *Ncoa4* deletion markedly increased STAT3 activity, as measured by a Sis-Inducible Element (SIE) luciferase assay (**Fig. S7A**). Pharmacological inhibition of MCU with minocycline significantly reduced phosphorylated STAT3 (p-STAT3) expression (**Fig. S7B**) and suppressed enhanced xenograft growth driven by *Ncoa4* deficiency (**Figs. S7C, S7D**).

Furthermore, treatment with the STAT3 inhibitor NSC78956 or the iron chelator deferiprone (DFP) effectively attenuated tumor growth in *Ncoa4* deletion models (**Figs. S7E, S7F**). These results support a model whereby *Ncoa4* deletion promotes mitochondrial iron accumulation and oxidative stress via MCU upregulation, activating pro-tumorigenic signaling including STAT3.

### NCOA4 overexpression inhibits colorectal cancer progression via MCU-dependent mitochondrial iron regulation

**Figure 7:**
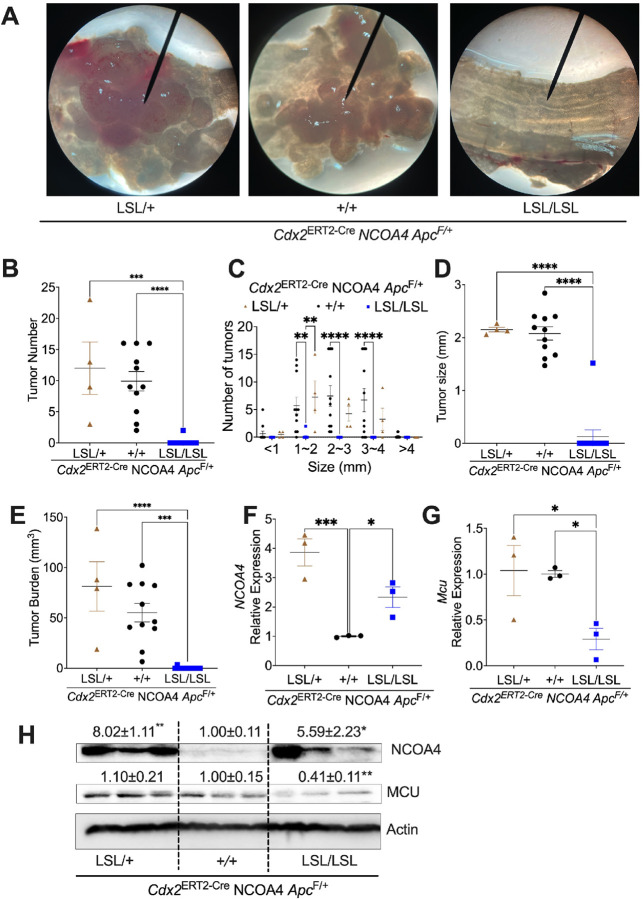
Homozygous overexpression of NCOA4 in mice represses colon tumor growth via MCU suppression. (**A**) Representative gross images; (**B**) tumor count; (**C**) tumor count based on size; (**D**) average tumor size; (**E**) tumor burden; qPCR analysis of (**F**) *NCOA4* and (**G**) *Mcu,* (**H**) Immunoblot analysis of colon tumors from *Cdx2*^ERT2-Cre^ *NCOA4*^LSL/+^ *Apc*^F/+^ (n = 3–4), *Cdx2*^ERT2-Cre^ *NCOA4*^+/+^ *Apc*^F/+^ (n = 3–11), and *Cdx2*^ERT2-Cre^ *NCOA4*^LSL/LSL^ *Apc*^F/+^ (n = 3–12). Values above blots are mean ± S.D. *p < 0.05, **p < 0.01, ***p < 0.001, ****p < 0.0001. Statistical analysis was performed using one-way ANOVA followed by Dunnett’s multiple comparisons test for B, D, E-H. Two-way ANOVA followed with Sidak’s multiple comparisons test for C.

To further validate NCOA4’s tumor-suppressive role via MCU regulation, we used a mouse model with inducible NCOA4 overexpression (*Cdx2*^ERT2-Cre^ *NCOA4*^LSL/+^ and *Cdx2*^ERT2-Cre^ *NCOA4*^LSL/LSL^ alleles) combined with *Apc*^F/+^ to induce colon tumors via TAM and DSS treatment. Gross colon images showed visibly fewer and smaller tumors in homozygous *NCOA4*^LSL/LSL^ mice compared to heterozygous or wild-type controls (Fig. 7A). Quantitative tumor counts and average tumor size were significantly reduced in the homozygous group (Figs. 7B–7D), with tumor burden also markedly decreased (Fig. 7E). qPCR and immunoblot confirmed increased *Ncoa4* mRNA and protein expression in *Cdx2*^ERT2-Cre^ *NCOA4*^LSL/+^ and *Cdx2*^ERT2-Cre^ *NCOA4*^LSL/LSL^ mice (Figs. 7F, 7H). Notably, only homozygous *Cdx2*^ERT2-Cre^ *NCOA4*^LSL/LSL^ mice exhibited decreased *Mcu* mRNA and protein levels (Figs. 7G, 7H), indicating a mechanistic link between NCOA4 overexpression and reduced mitochondrial iron uptake. Together, these results support that NCOA4 overexpression inhibits colon tumor development, at least in part, by downregulating MCU and modulating mitochondrial iron metabolism.

## Discussion

Our study uncovers key upstream mechanisms of iron-driven colorectal tumorigenesis and identifies novel therapeutic targets centered on the regulation of ferritinophagy. While previous reports have demonstrated that NCOA4-mediated ferritinophagy can suppress tumor growth in cancers such as pancreatic cancer and acute myeloid leukemia (Santana-Codina et al., 2022; Ravichandran et al., 2022; Larrue et al., 2024), our findings—consistent with a bioinformatic study linking lower NCOA4 levels to poorer prognosis in CRC (Gu et al., 2022)—reveal a contrasting role for NCOA4 in colorectal cancer. Specifically, we show that loss of NCOA4 increases both cytosolic and mitochondrial iron levels, thereby promoting CRC cell growth and supporting a pro-tumorigenic role for NCOA4 deficiency in the colorectal tumor microenvironment.

This work represents one of the first experimental validations of NCOA4 as a context-dependent oncogenic modifier in CRC, challenging the prevailing notion that NCOA4 inhibition is universally tumor-suppressive. Our results underscore the importance of evaluating the role of NCOA4 and ferritinophagy in a cancer-type–specific manner and highlight the therapeutic potential of targeting iron regulatory pathways in colorectal cancer.

Mechanistically, loss of NCOA4 impairs ferritinophagy, leading to iron sequestration and a cellular iron starvation response. This response drives the upregulation of iron uptake machinery, including the IRP2–TFRC axis and HIF-2α–DMT1/STEAP4 pathway, which collectively promote intracellular iron accumulation and CRC progression. Our extensive prior work (Xue et al., 2012, 2016, 2017; Schwartz et al., 2021; Kim et al., 2023; Villareal et al., 2024; Arcos et al., 2025) and that of others (Pusatcioglu et al., 2014; Radulescu et al., 2012) consistently demonstrate that CRC tumors accumulate iron to sustain growth and progression, reinforcing iron metabolism as a promising therapeutic target.

Importantly, our data reveal a novel link between NCOA4 deletion and mitochondrial iron overload, resulting in elevated mtROS. Proteomic analyses indicated that canonical mitochondrial iron transporters, mitoferrin-1 (MFRN1) and MFRN2, were not significantly upregulated. Instead, the mitochondrial calcium uniporter (MCU) emerged as one of the most highly expressed proteins associated with mitochondrial iron accumulation. This mitochondrial dysfunction appears to be driven by MCU upregulation, which facilitates not only calcium influx but also mitochondrial iron uptake (Zhang et al., 2019; Arcos et al., 2025). MCU overexpression and the resulting increase in mtROS serve as second messengers to activate downstream oncogenic pathways—most notably the iron- and ROS-dependent STAT3 signaling cascade (Xue et al., 2016; Cao et al., 2020). STAT3 activation is well established in cancer and linked to enhanced proliferation, survival, immune evasion, and metastasis (Lee et al., 2019; Wang et al., 2022; Li et al., 2025), further emphasizing the oncogenic potential driven by NCOA4 loss.

The reciprocal relationship between NCOA4 and MCU expression is particularly noteworthy. NCOA4 overexpression suppresses CRC tumorigenesis and correlates with reduced MCU levels, suggesting that NCOA4 maintains mitochondrial homeostasis by limiting MCU-mediated iron influx. This balance is critical, as mitochondrial iron and ROS levels are tightly connected to cellular bioenergetics, apoptosis, and redox signaling (Richardson et al., 2010; Nakamura and Takada, 2021; Zhao et al., 2024). Disruption of this equilibrium through NCOA4 deletion leads to metabolic reprogramming conducive to tumor growth (Liu et al., 2022). Future studies generating NCOA4 and MCU double knockout mice would be valuable to directly test MCU’s role in mitochondrial iron accumulation in the absence of NCOA4.

Our findings underscore the complex interplay between iron metabolism, mitochondrial function, and oncogenic signaling in CRC. They suggest that targeting iron uptake pathways, mitochondrial iron handling, or downstream effectors such as STAT3 could represent viable therapeutic strategies, especially in tumors characterized by NCOA4 loss or low expression. Therapeutic approaches using iron chelators or MCU inhibitors—alone or combined with STAT3 pathway antagonists—warrant further investigation to counteract NCOA4 deficiency–driven tumorigenesis.

Our study also indicates that ferritinophagy actively maintains iron homeostasis in CRC cells, with the observed phenotypes likely reflecting long-term consequences of NCOA4 ablation. It would be valuable to assess effects at earlier time points post-NCOA4 deletion—using inducible knockdown systems—to determine whether acute NCOA4 loss produces similar or distinct outcomes on cell growth and iron metabolism. Specifically, it will be important to examine if acute NCOA4 ablation transiently decreases cytosolic iron, triggering compensatory TFRC upregulation and possibly resulting in an overshoot toward iron overload.

In summary, our study elucidates a tumor-promoting function of NCOA4 loss in colorectal cancer through dysregulation of iron homeostasis and mitochondrial dynamics. These insights reveal novel molecular targets and pathways for therapeutic development, which may ultimately improve outcomes for patients with NCOA4-deficient colorectal cancers.

## Materials and Methods

### Cell Culture

Human HCT116 (RRID: CVCL_0291) and mouse MC38 (RRID: CVCL_B288) colorectal cancer cell lines were maintained at 37°C in a humidified atmosphere containing 5% CO_2_. Cells were cultured in Dulbecco’s Modified Eagle Medium (DMEM, Cat. #11965092, Thermo Fisher Scientific, Waltham, MA) supplemented with 10% fetal bovine serum (FBS, Cat. #F2442–500ML, Millipore Sigma, Burlington, MA) and 1% penicillin-streptomycin (10,000 U/mL; Cat. #15140122, Thermo Fisher Scientific). To generate stable NCOA4 knockout cell lines (sg*NCOA4*), HCT116 and MC38 cells were transfected with px459-NCOA4 or empty control plasmids using Lipofectamine 2000 (Cat. #11668027, Thermo Fisher Scientific). After transfection, cells were selected with puromycin to establish knockout clones.

### Animals

Animal studies were conducted to address specific research questions, adhering to the Institute of Laboratory Animal Resources guidelines, and approved by the Institutional Animal Care and Use Committee (IACUC) at the University of New Mexico Health Sciences Center (Protocol# 23–201434-HSC), following the National Institutes of Health guide for the care and use of laboratory animals (NIH Publications No. 8023, revised 1978). Mice, encompassing both sexes, were housed in standard cages under a 12-h light–dark cycle, in a temperature-controlled environment, with ad libitum access to a standard chow diet and water unless otherwise specified.

Colon-specific NCOA4 knockout and overexpression mouse models were generated by crossing *Ncoa4* floxed (*Ncoa4*^F/F^) and *NCOA4*^LSL/LSL^ mice (Santana-Codina et al., 2022) with tamoxifen-inducible *Cdx2*^ERT2 − Cre^ mice (Yin et al., 2025). These crosses produced *Cdx2*^ERT2 − Cre^ *Ncoa4*^F/F^ and *Cdx2*^ERT2 − Cre^ N*COA4*^LSL/LSL^ mice, allowing selective deletion or overexpression of NCOA4 in the colon epithelium upon tamoxifen treatment.

Colorectal cancer (CRC) was modeled using two established approaches. In the colitis-associated cancer (CAC) model, mice received intraperitoneal injections of azoxymethane (AOM) at 1 mg/mL to deliver a total dose of 10 mg/kg over two consecutive days. Beginning the day after AOM injection, tamoxifen was administered at 100 mg/kg for three consecutive days to induce NCOA4 deletion. Following tamoxifen treatment, mice were given 2% dextran sodium sulfate (DSS) in drinking water for seven days to induce colonic inflammation, followed by 14 days of regular water for tissue recovery. This inflammatory and recovery cycle was repeated once. In the second model, CRC was induced by combining monoallelic *Apc* deletion with DSS treatment. *Cdx2*^ERT2 − Cre^ *Apc*^F/+^ mice, *Cdx2*^ERT2 − Cre^ N*coa4*^F/F^ *Apc*^F/+^ mice and *Cdx2*^ERT2 − Cre^ *NCOA4*^LSL/LSL^ *Apc*^F/+^ mice were generated. After tamoxifen-induced recombination, mice received 2% DSS in drinking water for seven days, followed by 28 days of recovery with regular water. These models allowed assessment of NCOA4’s role in genetically predisposed and inflammation-driven tumorigenesis.

For *in vivo* tumor growth studies, MC38 murine CRC cells with stable NCOA4 knockout were injected subcutaneously into the flanks of syngeneic C57BL/6 mice at 1 × 10^6^ cells per site. Tumors were harvested after two weeks. For pharmacological inhibition studies, treatment began one week after injection once tumors were palpable. Mice were treated with vehicle control, minocycline at 20 mg/kg daily, NSC74859 (a STAT3 inhibitor) at 5 mg/kg intraperitoneally every other day, a Vitamin E-deficient diet (TD.88163, Envigo), a Vitamin E control diet (50 IU, TD.99455, Envigo), or deferiprone (DFP) at 1 mg/mL in drinking water. Treatments lasted for one week.

### Histology, Immunofluorescence, and DAB-Enhanced Perl’s Iron Staining

Formalin-fixed, paraffin-embedded colon tissue sections were processed for hematoxylin and eosin (H&E) staining, immunofluorescence (IF), and iron staining. For H&E staining, sections were deparaffinized and rehydrated through graded ethanol to distilled water. Sections were stained with hematoxylin for two minutes, rinsed in tap water, dipped briefly in bluing solution, and rinsed again for two minutes. Eosin staining was performed for five minutes, followed by dehydration through ethanol series, clearing in xylene, and mounting for histopathological evaluation by a gastrointestinal pathologist (Dr. Martin) in a blind manner.

For immunofluorescence, antigen retrieval was performed by incubating sections in 10 mM sodium citrate buffer at sub-boiling temperature for 12 minutes. Slides were cooled to room temperature for two hours and then blocked with 10% normal goat serum in 0.1% Triton X-100 in phosphate-buffered saline (PBS) or Tris-buffered saline (TBS) for one hour. Primary antibodies were diluted in 1% normal goat serum with 0.1% PBST or TBST and incubated overnight at 4°C. After washing, fluorophore-conjugated secondary antibodies diluted in 1% normal goat serum were applied for one hour at room temperature. Slides were washed and mounted using EverBrite^™^ Mounting Medium (Biotium). Primary antibodies used included cleaved Caspase-3 (CC3, #9664), Ki-67 (#12202), and phospho-STAT3 (p-STAT3, #9145) from Cell Signaling Technology.

### FerroOrange, Mito-Ferro Green, DCFH-DA, and MitoSOX Staining

Cells were seeded at 2 × 10^5^ per well in 24-well plates. After adherence, cells were stained in pre-warmed Hank’s Balanced Salt Solution (HBSS) containing fluorescent probes: 0.5 μM FerroOrange for cytosolic ferrous iron (Dojindo), 1 μM Mito-Ferro Green for mitochondrial iron, 10 μM 2’,7’-dichlorodihydrofluorescein diacetate (DCFH-DA; Cayman Chemical) for total cellular reactive oxygen species (ROS), and 1 μM MitoSOX (Thermo Fisher Scientific) for mitochondrial superoxide. Staining was performed at 37°C for 30 minutes, followed by washing with PBS. Fluorescence images were captured using an Invitrogen^™^ EVOS^™^ FL Auto Imaging System. The RFP channel was used for FerroOrange and MitoSOX, while the GFP channel was used for DCFH-DA and Mito-Ferro Green. ImageJ software was used to quantify fluorescence intensity normalized to Hoechst 33342 nuclear staining.

### Luciferase reporter gene assay

To measure luciferase activity, cells were seeded at 5 × 10^4^ per well in 24-well plates and transfected using polyethylenimine (PEI) with either the pGL4.47[luc2P/SIE-RE/Hygro] reporter plasmid containing a STAT3-responsive element alongside a LacZ plasmid for normalization, or the human NQO1-ARE TATA-Inr luciferase reporter plasmid (for MC38 wild-type and sgNcoa4 cells, Yin et al., 2025). After 48 hours, cells were lysed in lysis buffer. Luciferase activity was then quantified using a Promega Luciferase Assay Kit (E1500), with 20 μL of supernatant added to a 96-well plate and 100 μL of luciferase reagent per well. Luminescence intensity was measured by a plate reader and normalized to protein concentration.

### Quantitative Polymerase Chain Reaction (qPCR) Analysis

Total RNA was extracted using the IBI Isolate DNA/RNA Reagent Kit following manufacturer’s instructions. For RNA from DSS-treated colon tissues, an additional purification was performed by precipitating RNA with 8 M lithium chloride (LiCl), followed by ethanol washes to improve RNA quality. RNA was resuspended in DEPC-treated water and quantified. Quantitative PCR was conducted using the LightCycler 480 system (Roche Diagnostics) with gene-specific primers listed in **Table S2**. Relative gene expression was calculated by the ΔΔCt method using housekeeping genes for normalization.

### Immunoblotting Analysis

Cells and tumor tissues were lysed in radioimmunoprecipitation assay (RIPA) buffer. Lysates were centrifuged, and protein concentration was determined using a BioTek Synergy HTX Multi-Mode Microplate Reader. Equal amounts of protein (10–50 μg) were loaded on SDS-PAGE gels, separated by electrophoresis, and transferred to nitrocellulose membranes. Membranes were blocked with 5% milk and incubated overnight with primary antibodies, followed by incubation with appropriate secondary antibodies for 1.5 hours. The antibodies used are listed in **Table S3**.

### ATP Measurement

Cells (5 × 10^3^) were seeded in white opaque 96-well plates. After 48 hours, an equal volume of CellTiter-Glo^®^ 2D Cell Viability Assay reagent was added. Luminescence was measured after 10 minutes using a SpectraMax M5 Microplate Reader.

### MTT Assay

Cells were seeded at 5 × 10^4^ cells/mL in 24-well plates. After treatments, 125 μL of 5 mg/mL MTT solution was added and incubated at 37°C for 30 minutes. Formazan crystals were dissolved in DMSO, and absorbance at 570 nm was measured with a BioTek Synergy HTX Microplate Reader.

### Inductively Coupled Plasma Mass Spectrometry (ICP-MS) Analysis

Tissue samples were digested overnight in concentrated nitric acid and diluted with Milli-Q water. Samples were analyzed on an Agilent 7900 ICP-MS instrument at the University of New Mexico Health Sciences Center. Iron content was normalized to tissue weight.

### Unbiased Quantitative Proteomics Study

*Ncoa4*^F/F^ and *Cdx2*^ERT2 − Cre^ *Ncoa4*^F/F^ mice were treated with tamoxifen (100 mg/kg daily for three days) to induce colon-specific Ncoa4 deletion. One week after the final injection, mice were sacrificed, and colonic epithelial cells were isolated by scraping the luminal surface. Proteomic analysis was performed as described previously (Santana-Codina et al., 2022).

### Statistical Analysis

Data are presented as mean ± standard deviation. Statistical significance was evaluated using independent or paired t-tests, one-way or two-way ANOVA where appropriate. A p-value of less than 0.05 was considered statistically significant.

## Supplementary Files

This is a list of supplementary files associated with this preprint. Click to download.

- TableS1XueProteomedataNSC.xlsx
- 250806SupplementalTable2and3.docx
- 250809SupplementaryfiguresV1.docx

## Data Availability

All data relevant to the study are included in the article or uploaded as online supplemental information. Additional data are available from the corresponding author upon reasonable request.
